# Factors Influencing Pseudo-Accommodation—The Difference between Subjectively Reported Range of Clear Focus and Objectively Measured Accommodation Range

**DOI:** 10.3390/vision3030034

**Published:** 2019-06-28

**Authors:** Sandeep K. Dhallu, Amy L. Sheppard, Tom Drew, Toshifumi Mihashi, Juan F. Zapata-Díaz, Hema Radhakrishnan, D. Robert Iskander, James S. Wolffsohn

**Affiliations:** 1 Optometry and Vision Science Research Group, Life and Health Sciences, Aston University, Birmingham B4 7ET, UK; 2 Department of Ophthalmology, Faculty of Medicine, University of Tsukuba, Tsukuba 305-8575, Japan; 3 Department of Clinical Research, Vista Ircovisión Oftalmólogos, 30008 Murcia, Spain; 4 Faculty of Biology, Medicine and Health, The University of Manchester, Manchester M13 9PL, UK; 5 Department of Biomedical Engineering, Wroclaw University of Science and Technology, 50-370 Wroclaw, Poland

**Keywords:** subjective range of focus, objective accommodation, depth of focus, aberrations, pupil size, tolerance to blur, presbyopia

## Abstract

The key determinants of the range of clear focus in pre-presbyopes and their relative contributions to the difference between subjective range of focus and objective accommodation assessments have not been previously quantified. Fifty participants (aged 33.0 ± 6.4 years) underwent simultaneous monocular subjective (visual acuity measured with an electronic test-chart) and objective (dynamic accommodation measured with an Aston open-field aberrometer) defocus curve testing for lenses between +2.00 to −10.00 DS in +0.50 DS steps in a randomized order. Pupil diameter and ocular aberrations (converted to visual metrics normalized for pupil size) at each level of blur were measured. The difference between objective range over which the power of the crystalline lens changes and the subjective range of clear focus was quantified and the results modelled using pupil size, refractive error, tolerance to blur, and ocular aberrations. The subjective range of clear focus was principally accounted for by age (46.4%) and pupil size (19.3%). The objectively assessed accommodative range was also principally accounted for by age (27.6%) and pupil size (15.4%). Over one-quarter (26.0%) of the difference between objective accommodation and subjective range of clear focus was accounted for by age (14.0%) and spherical aberration at maximum accommodation (12.0%). There was no significant change in the objective accommodative response (F = 1.426, *p* = 0.229) or pupil size (F = 0.799, *p* = 0.554) of participants for levels of defocus above their amplitude of accommodation. Pre-presbyopes benefit from an increased subjective range of clear vision beyond their objective accommodation due in part to neural factors, resulting in a measured depth-of-focus of, on average, 1.0 D.

## 1. Introduction

The subjectively experienced range of clear focus during accommodation is greater than the objectively measured increase in ocular power due to the depth of focus [1,2,3,4]. Accommodation is widely accepted to be achieved by a change in shape of the crystalline lens secondary to ciliary muscle contraction [1,5,6,7,8,9]. However, pseudophakic patients implanted with monofocal intraocular lenses (IOLs) are sometimes able to demonstrate relatively good near ability through non-accommodative means, attributed to the change in pupil size, anterior IOL movement, higher order aberrations (HOAs), and tolerance to blur [3,8,10,11,12,13]. In addition, vergence and spatiotopic cues drive accommodation [14], which can also be influenced by voluntary control [15].

Blur tolerance is known to increase with age [16,17] as a result of age-related optical factors, specifically pupil miosis, as well as experience-mediated neural compensation [18]. A study investigating why older subjects were better able to read optically blurred text than younger subjects found that neural adaptation from long term visual experience played an important part in the observed superior reading ability of older subjects [18]. Blur thresholds increase with a smaller pupil size [19] and with greater retinal eccentricity [20,21], the latter thought to occur due to a combination of anatomical, physiological, optical and perceptual factors such as sharpness overconstancy [21,22]. HOAs arise from imperfections in the eye’s structure, particularly the cornea and crystalline lens, and influence the retinal image quality [23,24,25,26,27]. They are dynamic and change with pupil size, age and accommodation [28,29,30,31,32]. HOAs such as spherical aberration are thought to act as cues for best focus and can increase the depth-of-focus (DoF) [6]. Similarly, chromatic effects from longitudinal chromatic aberration may help direct the eye’s focusing system [33].

Subjective DoF describes the range of image distances in front of and behind the focal point over which the image is perceived as being in focus without causing any objectionable reduction in image sharpness [6,8], thus providing a perceptual tolerance for small errors in ocular focus [34]. Subjective DoF is reduced by increasing target luminance [19,34,35], contrast [19,34,35,36], spatial frequency [5,16] and detail [2,5,36,37,38] as well as patient visual acuity [16,39], pupil size [2,19,35,36,40] and blur sensitivity [21,34], whereas it increases with increasing chromatic aberration [34] and retinal eccentricity [34,41].

Pseudoaccomodative factors such as pupil miosis and aberrations can increase the subjective DoF [11,42,43,44]. Also, accommodation close to the maximum accommodative amplitude can be sustained for prolonged periods. Thus, attempts to restore focus may not require the full level of accommodation demanded by the target vergence [45]. This may in part explain the relatively high circumstantial satisfaction with ‘accommodating’ intraocular lenses, despite the apparent lack of significant objective accommodation [10,11,42,46,47].

Although pupil size, HOAs, and tolerance to blur are thought to cause the observed difference between the subjective range of clear focus and objective accommodation measurements, the amount of the variance explained and the relative contribution of each factor to the observed difference is currently unknown. Therefore, the purpose of this study was to investigate the factors affecting the subjective range of clear focus (compared to the objective accommodation) in pre-presbyopes and to determine the relative contribution of these factors to the difference between them.

## 2. Materials and Methods

All subjects gave their informed consent for inclusion before they participated in the study. The study was conducted in accordance with the Declaration of Helsinki, and the protocol was approved by the Ethics Committee of Aston University (Project identification code #606). To take part in this study, participants were required to: be aged between 20 and 45 years, have no more than 0.75 dioptres (D) of uncorrected astigmatism, be able to wear contact lenses, have a corrected visual acuity of ≤0.00 logMAR, be free of any active eye disease, not be taking ocular medications or systemic medications with known ocular side effects, and to have no history of eye surgery.

Fifty participants, naïve to visual testing, underwent subjective refraction at 4 m by the same Optometrist (S.K.D.) during which plus power was maximized, while maintaining the participants’ best visual acuity. The participants’ mean spherical equivalent distance refraction was then corrected with contact lenses. After a settling time of at least 30 min while participants rested, subjective amplitude of accommodation was measured using a push-up test that was repeated three times and averaged [48]. During testing, participants were directed to the smallest print on the Royal Air Force (RAF) rule that could be seen clearly when the slider was at the furthest end of the rule and subjective range of clear focus was calculated by averaging the difference between the first reported point of unresolvable blur on push-up and the first point blur could be resolved on push-down [48].

**Figure 1:**
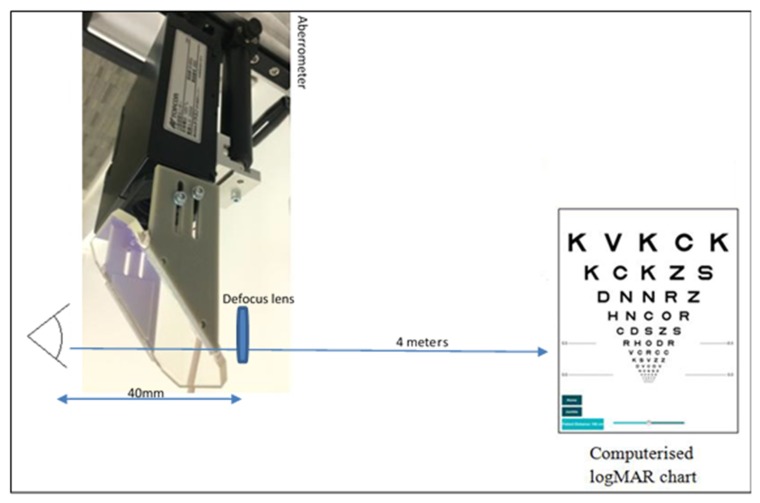
Schematic. Diagram illustrating the study set-up.

A defocus curve is a measure of visual acuity at different distances or with different levels of trial lens induced defocus and is used to evaluate range of clear vision [49,50]. Monocular subjective defocus curves were measured with the participant seated, and, at the same time objective aberrometry images were captured to assess accommodation. Full aperture trial lenses from +2.00 to −10.00 DS in +0.50 steps were placed in front of the eye, but outside of the aberrometer measurement path, in order to alter the focal demand for viewing a distance object [49,51]. Trial lenses were presented in a randomized order with a thirty second gap maintained between each lens presentation, in order to minimize the chance of the previous trial lens affecting the visual acuity measurement with subsequent lenses [52,53]. All subjective acuities were corrected for magnification effects associated with the lens power and back vertex distance [50]. Objective measurements of ocular aberrations (up to the 8th radial order of Zernike polynomials) and also pupil size at each level of defocus were acquired using the Aston open field Shack-Hartmann aberrometer [54], with participants viewing and reading the smallest visible letters from the 4 m distance logMAR chart (TestChart 2000Pro, Thomson Software Solutions, Hatfield, UK) through the instruments beam splitter (Figure 1). The letters were randomized between each presentation in order to reduce learning effect [49,50], with each correctly read letter scored as 0.02 logMAR; participants were encouraged to guess if unsure. A validation study comparing the Aston aberrometer with a conventional aberrometer found a mean difference of 0.02 D ± 0.49 D [95% confidence interval] in mean spherical equivalent (MSE) and excellent intrasession repeatability (MSE = 1.000, *p* < 0.001) [54]. Simultaneously with subjective vision assessment, the aberrometer captured aberrations centered on the pupil, and over the whole of the pupil area as well as pupil diameter at each level of set defocus. The simultaneous subjective defocus curve and objective aberrometry measurements were taken in identical lighting conditions for all participants. Trial lenses were positioned 40 mm from the corneal plane, so they were not in the aberrometer path and were powered to create the traditional defocus step sizes of 0.50 D at a back-vertex distance equivalent to 12 mm.

**Figure 2:**
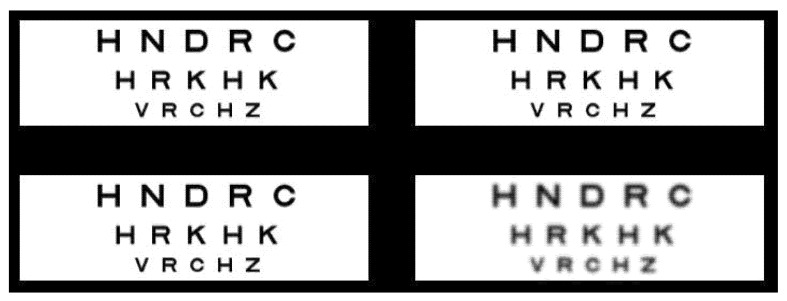
Tolerance to blur visual target, with the blurred target in the bottom right hand quadrant in this example.

Blur tolerance was assessed with a 4 alternate-forced choice (AFC) spatial task in which the participant was required to identify the one target that differed from the others [20]. A study comparing a 2, 4 and 8 AFC method found that naïve observers showed the highest sensitivity and reliability with a 4 AFC test for detection tasks [55]. The visual target used comprised of four shortened logMAR charts (−0.1, 0.0 and 0.2 lines of >95% contrast black letters against a white background) located in each quadrant of the screen. A shortened logMAR chart was chosen to minimize cues, such as the blurred edges of larger letters or the reduced contrast of smaller letters, to aid in the subjective assessment of blur. As the target size increases, blur thresholds increase and so blur sensitivity decreases; therefore a target close to the limit of the participant’s acuity was selected [19]. GIMP open source software (version 2.6, https://www.gimp.org/) was used to blur one of the test charts selected at random by differing levels using a Gaussian blur filter, with a higher filter value producing a higher amount of blur. Convolution was used to low-pass the image, with the sigma input parameter set as the standard deviation of the Gaussian, in pixels [48]. Participants were asked to view the high contrast, illuminated (85 cd/m^2^), computerized visual target monocularly, which was placed two meters away, through a subjectively aligned 1.5mm pinhole in order to neutralize aberration and pupil size effects. Participants were given 30 seconds to identify the location of the blurred chart. After a familiarization demonstration (with a 5.5 Gaussian filter), subsequent plates had decreasing amounts of Gaussian blur applied logarithmically in 1.2 log steps in a double reversal staircase procedure to one of the 4 logMAR charts, with participants asked to select which quadrant this occurred in (Figure 2). A logarithmic blur progression was adopted to provide a balance between accurate blur discrimination assessment and testing time [56]. A featureless black plate was displayed for 5 seconds between each test plate page to allow participants to recover before seeing the next image, and also to stop participants from potentially identifying the blurred logMAR chart by the change in letter clarity from one plate to the next.

A Matlab (The Mathworks, Natick, MA, USA) script was used to model the participant’s objective DoF from wavefront pupil size measurements. This script was used to calculate the image quality (IQ) at all defocus levels. The metric used to calculate the IQ was the augmented visual Strehl ratio based on the optical transfer function (VSOTFa) [57].

(1) VSOTFa=∫−∞∞∫−∞∞CSFN(fx,fy)·|Re{OTF(fx,fy)}|dfxdfy∫−∞∞∫−∞∞CSFN(fx,fy)·OTFDL(fx,fy)dfxdfy

where ${\mathrm{OTF}}_{\mathrm{DL}}$ is the diffraction limited optical transfer function, ${\mathrm{CSF}}_{N}$ is the neural contrast sensitivity function and $\left(f_{x},f_{y}\right)$ are the spatial frequency coordinates [56]. The augmented visual Strehl ratio overcomes many of the limitations of the original such as its complexity, potential negativity or value >1 and sensitivity to the presence of prisms in the wavefront aberration, resulting in a metric that correlates better with visual performance [57].

**Figure 3:**
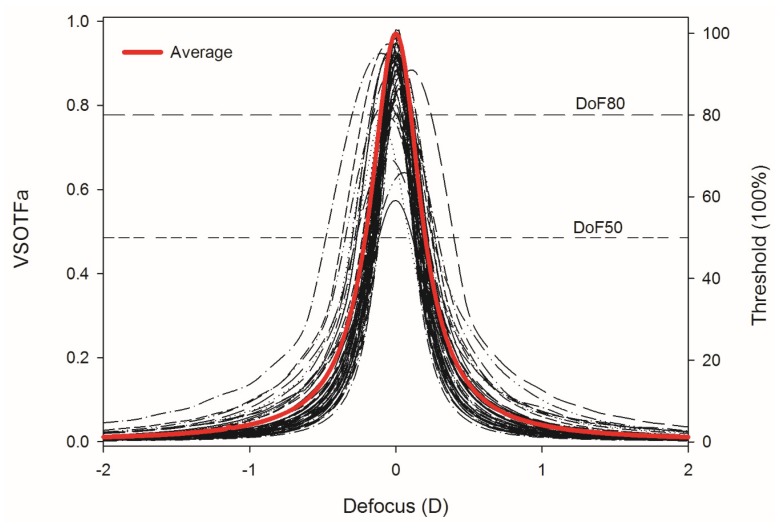
Through-focus VSOTFa analysis of each participant at maximum accommodation. Dashed lines represent 50% and 80% thresholds of the maximum IQ. DoF50 and DoF80 are the estimated values of objective DoF for the 50% threshold and the 80% threshold respectively.

Retinal image-based metrics are considered good predictors of subjective visual performance [58,59]. The criterion used to model the objective DoF was the range of defocus over which the IQ does not fall below a certain relative threshold. This method has been previously used and fully described in Yi et al. (2010) [60]. Previous studies have chosen fixed thresholds of 50% [16,42] and 80% [5] of the maximum image quality to calculate DoF range (Figure 3). Both thresholds (50% and 80%) were used in this study and the analysis was performed for maximum accommodation.

**Figure 4:**
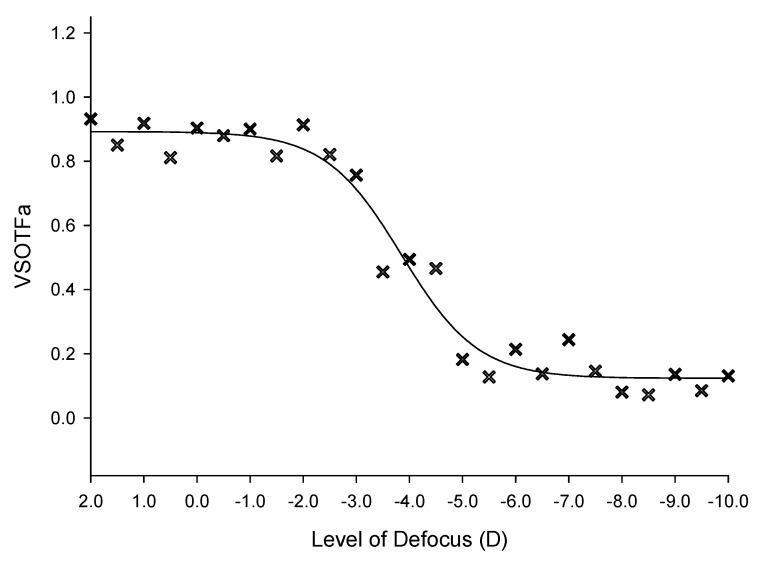
Plot of the sigmoidal fit of VSOTFa IQ metric. One participant’s normalized image quality visual metric to which curve fitting was applied (solid line) in order to determine the initial point of plateau.

The objective defocus range was then calculated for each participant using dynamic curve fitting from the VSOTFa normalized for pupil size at each defocus level (SigmaPlot v11.0, Systat Software Inc., Chicago, IL, USA) to locate the initial point of plateau. A sigmoidal equation was selected to fit the data dynamically in an iterative process to minimize the sum of squared errors (see an example in Figure 4). Absolute values for the subjective range of clear focus (blur level at which the visual acuity became worse than 0.3 logMAR—equivalent to the driving standard in many countries) were calculated for each participant to the nearest 0.1 D [50].

Sample size estimation revealed that a minimum of 47 participants were required to achieve a power of 80% for a correlation coefficient of 0.4 with a significance level of 0.05 (Version 11, Systat Software Inc., Chicago, IL, USA). A one-sample Kolmogorov-Smirnov test did not reject the hypothesis that the data was normally distributed (*p* > 0.05), therefore parametric analysis was used. Pearson’s correlations were used to examine the relationship between variables, and analysis of variance for changes in pupil diameter and objective accommodation with lens induced defocus blur. Stepwise forward linear regression analysis (F entry at *p* < 0.05 and removal at *p* > 0.10) was performed in order to identify the key factors influencing the difference between subjective and objective measures and to determine their relative contribution to this difference (adjusted *r*^2^). Due to the confounding effect of intercorrelation on the modelling, only significant factors in the correlation matrix were included.

## 3. Results

The fifty participants had a mean age 33.0 ± 6.4 years (range 23–45 years; 23–25 years: *n* = 5, 26–30 years: *n* = 17, 31–35 years: *n* = 10, 36–40 years: *n* = 9, and 41–45 years: *n* = 9; 30 females). The group had a mean spherical equivalent refraction error of -1.48 D (ranging from -14.00 D to +0.50 D) with a mean cylindrical refraction of −0.75 D (ranging from 0 D to −0.75 D).

**Figure 5:**
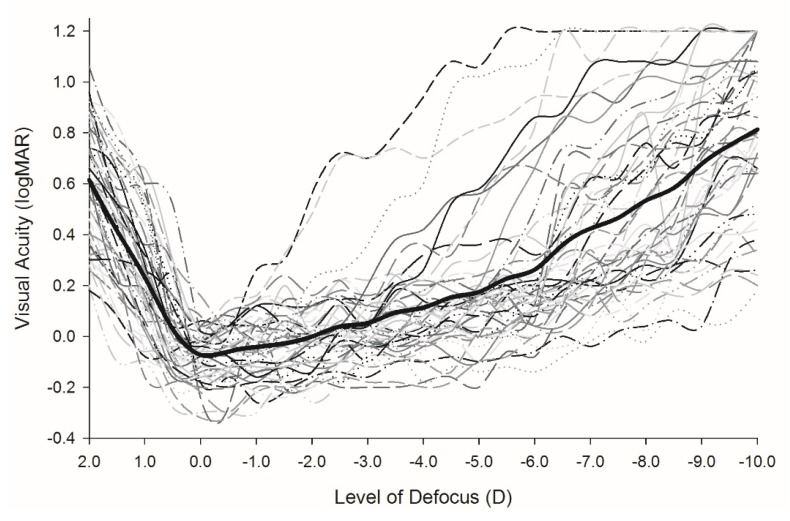
Subjective defocus curves. Visual acuity of each participant with the level of optical defocus. Thicker black line indicates mean values. *n* = 50.

**Figure 6:**
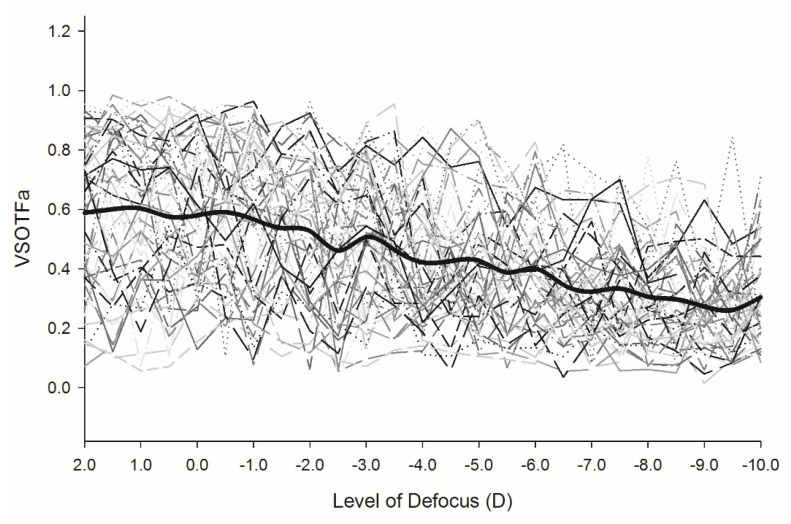
Objectively measured accommodative response converted to an image metric (VSOTFa) of each participant with the level of optical defocus. *n* = 50.

The subjective defocus curves are presented in Figure 5 and the objective accommodation VSOTFa IQ decrease followed by a plateau in Figure 6. The average amplitude of accommodation measured using the absolute criterion from subjective defocus curves was 7.7 ± 1.9 D, while the mean amplitude obtained from the subjective push-up test was 6.7 ± 1.7 D with a correlation of *r* = 0.803, *p* < 0.001). The average objective range of accommodation from the point of plateau of the VSOFTa IQ metric was 6.7 ± 1.9 D, which was lower than both subjective measurements in all participants, but correlated reasonably strongly with the push-up test (*r* = 0.728, *p* < 0.001). The average difference between the subjective range of clear focus and objective accommodative response (the measured DoF at maximum accommodation) was 1.0 ± 0.8 D.

The correlation between the subjective range of clear focus, objective accommodative range and the measured DoF with age, pupil diameter metrics, refractive error, tolerance to blur, ocular aberrations, and objective DoF modelling are presented in Table 1.

Age was strongly positively correlated to the subjective range of clear focus and objective accommodation as expected (Table 1). Pupil diameter was strongly negatively correlated with objective DoF modelling due to the fact that the optical image quality metrics used to model DoF take into account both ocular aberrations and pupil size. Tolerance to blur was on average 1.6 ± 1.0 D, but was not associated with any of the factors measured. Objective DoF modelling with both 50% and 80% thresholds was negatively correlated with the subjective range of clear vision and objective accommodation, but not the difference between them.

Pupil diameter decreased with increasing accommodating effort (5.05 mm ± 1.08 with distance viewing compared to 3.57 ± 1.08 mm when viewing a 10 D target, *p* < 0.001; ANOVA F = 60.70, *p* < 0.001), which along with a negative shift in fourth order spherical aberration (from 0.013 ± 0.012 µm in the unaccommodated state, to −0.019 ± 0.015 µm at maximum accommodation; *p* = 0.046), resulted in an increased modelled objective DoF (*p* < 0.001) for 50% (0.38 ± 0.12 vs 0.73 ± 0.29 respectively) and for 80% (0.19 ± 0.06 vs 0.40 ± 0.17 respectively). Refractive error did not correlate significantly with any of the parameters examined (*p* > 0.05).

Principal component analysis with varimax rotation confirmed that age, tolerance to blur and higher order aberrations were independent factors so would not confound the regression analysis modelling. Subjective range of clear focus was principally accounted for by age (46.4% of the variance), with an additional 19.3% determined by pupil size accounting for 65.7% of the variance in total (F = 29.627, *p* < 0.001). Objectively assessed accommodation range was also principally accounted for by age (27.6% of the variance) with an additional 15.4% determined by pupil size, accounting for 43.0% of the variance in total (F = 11.693, *p* < 0.001). The difference between the subjective range of clear focus and objective accommodation was associated with the patient’s age (14.0%) and spherical aberration at maximum accommodation (12.0%), accounting for 26.0% of the variance in total (F = 5.459, *p* = 0.090).

**Figure 7:**
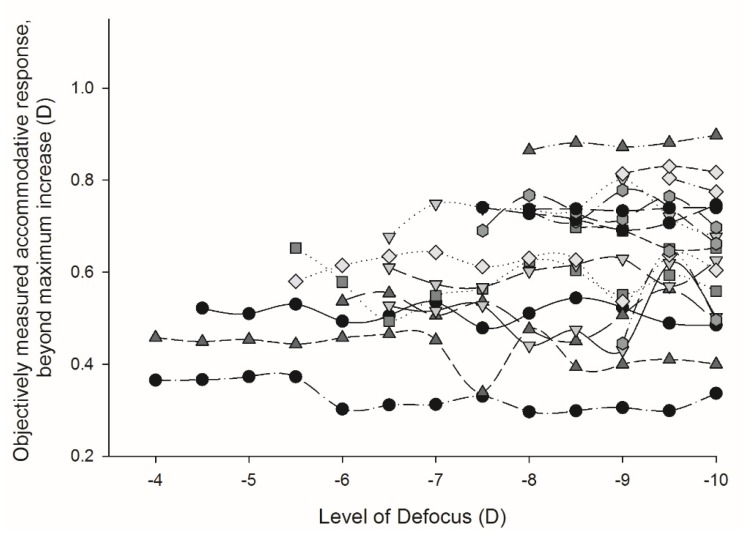
Objectively measured accommodative response, beyond maximum accommodation stimulation. The objectively measured accommodative response, once maximum accommodation had been stimulated for participants whose objective accommodative range was ≤9.5 D. *n* = 19, each symbol represents a participant’s response.

**Figure 8:**
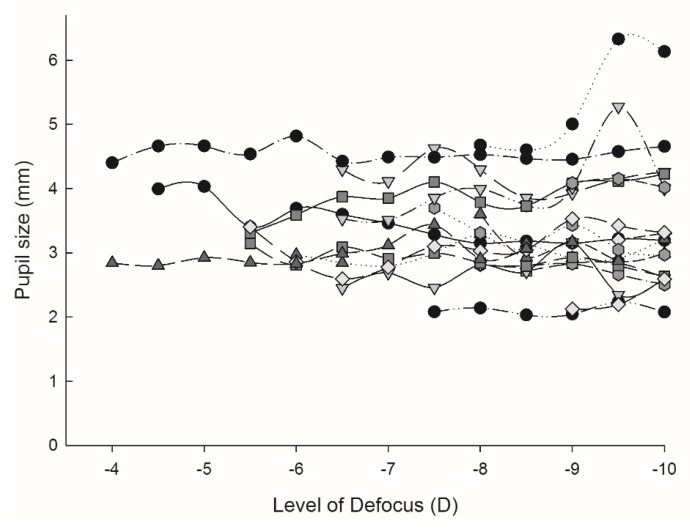
Pupil size, beyond maximum accommodation stimulation. The pupil size at each defocus lens once maximum accommodation had been stimulated *n* = 19, each symbol represents a participant’s pupil size.

The 19 participants that had reached their maximum objective accommodation by 9.5 D were instructed to continue focusing on the target as best they could through the remaining higher-powered defocus trial lenses. The objectively measured accommodative response after this point was then evaluated to determine the effect of the resultant blur on their accommodative response. A repeated measures analysis of variance (ANOVA) showed there to be no statistically significant difference in the accommodative response (ANOVA: F = 1.188, *p* = 0.363; Figure 7) or pupil size (ANOVA: F = 0.799, *p* = 0.554; Figure 8) of participants once maximum accommodation had been stimulated.

## 4. Discussion

Although pupil size, HOAs and tolerance to blur are thought to cause the observed difference between subjective range of clear focus and objective accommodation measurements [3,8,10,11,17,44,58,60], the amount of the variance explained and the relative contribution of each factor to the observed difference has not been previously determined. This range of clear focus is relevant to a presbyopic person with minimal residual objective accommodation, and those patients who are pseudophakic, such as following cataract surgery. The standard test for assessing accommodative range (push-up) correlated well with both subjective (defocus curve) and objective (optical change) methods, confirming this to be a reasonable assessment method in a clinical setting.

Although there is no standardized technique to assess a patient’s tolerance to blur, the technique adopted quantified this subjective neural phenomenon [18] largely independent of pupil size and ocular aberrations by viewing through a pinhole. Another approach would have been to use a closed-loop adaptive optics system to permit assessment of the neural blur tolerance that is essentially independent of optics, although scattering of light is not compensated with adaptive optics systems. Pupil diameter is known to affect the DoF of the eye [2,19,35,36] and while that was evident in the VSOTFa metric which uses pupil size in its calculation, neither pupil size nor VSOTFa were associated with the difference between subjective range of clear focus and objective accommodation. This could be due to the relatively small range in pupil size between participants (3.57 ± 1.08 mm when viewing the 10 D target) providing a relatively constant contribution to the range of clear focus. Although individual aberrations contribute significantly to the change in DoF of the eye [4,24,25,26,27], they are dynamic and change with pupil size, age and accommodation [28,29,30,31,32,44] and the interaction between different aberration terms can significantly increase or decrease the resultant DoF [23].

In this experimental design, the target luminance, spatial frequency and contrast were constant as these factors are known to affect the DoF [2,5,16,19,21,35,36,38] and can account for subjective performance in distance and near tasks in real-life settings. Participants aged between 23 and 45 years were examined as residual accommodation was required and a positive correlation was found between age and subjective range of clear focus or objective accommodation (Table 1). However, within this limited age group, there was no significant correlation between age and pupil size, aberrations, refractive error or tolerance to blur suggesting other ageing factors may contribute. About one-quarter of the variance between subjective range of clear focus and objective accommodation could be accounted for by age and spherical aberration at maximum accommodation. Microfluctuations of accommodation are known to increase with decreasing viewing distance so this may have added additional noise to the subjective judgements and objective measurement of aberrations [22]. Tonic accommodation may also play a role. While subjective variability will always limit the amount of variance that can be explained and the model considers all the inputs to be linear, this still suggests that further as yet undetermined factors contribute to this phenomenon. A recent paper has shown that less than 5% of the variance in the inter-individual differences in subjective amplitude of accommodation other than age, can be accounted for by optical factors, supporting this conclusion [61].

It should be noted that monocular blur driven accommodation is generally lower that binocular proximity driven accommodation [62], but the scaled dynamics are similar. However, objective and subjective amplitudes were measured under the same conditions simultaneously, so the relative differences are unlikely to be affected. Blur sensitivity has recently been found to be lower in myopes than emmetropes when viewing monocularly [63], but this was not observed in our cohort. Myopes have also been found to have greater accommodative lags than emmetropes and a more variable response [64], however non-cyclopleged refractive error (at least in the myopic range) was not found to be an influencing factor in the difference between subjective and objective accommodation.

The study showed that the maximum accommodation level was maintained in most participants beyond their point of blur and pupil size did not significantly alter. Hence studies measuring the maximum accommodation possible with new devices or techniques such as ‘accommodating’ IOLs or pharmaceutical agents [65] need only assess best corrected distance refraction and that at a close near distance in order to determine the maximum amount of physiologically driven accommodation possible. The DoF will aid the range of clear focus with such devices; hence, given a DoF on average of 1.0 D (subjectively tolerated defocus beyond objective accommodation) and considering that around eighty percent of the residual accommodation can be effectively used, even in sustained reading tasks [45] to read at 40 cm would require only 2.1 D of objective accommodation to be restored.

## 5. Conclusions

The difference between the subjective range of clear focus and objective accommodation (range of defocus demand over which the aberrations of the ocular system could respond) of approximately 1.03 ± 0.81 D was explained partly by age and spherical aberration at maximum accommodation, but this only explains around one-quarter of the variance, suggesting that other factors are yet to be identified. Pupil size and aberrations (incorporated in the visual metric modelling) contribute to the subjective range of clear focus and objective accommodative range, but would appear to provide a more constant contribution to the difference between them.

## Figures and Tables

**Table 1:** Relationship (Pearson’s correlation) between subjective and objective range of clear focus and age, pupil diameter, tolerance to blur, ocular aberrations and depth of focus modelling at maximum accommodation. *n* = 50. * indicates 2-tailed significance at *p* < 0.05 and ** at *p* < 0.01.

			Range of Clear Vision		Refractive Error	Tolerance to Blur	Ocular Aberrations		Age	Pupil Size	DoF Modelling	
			Objective	Difference			Higher order	Average spherical			50% (max accom)	80% (max accom)
**Range of Clear Vision**	**Subjective**	Pearson Correlation	0.910 **	0.237	0.126	0.021	−0.009	0.003	0.743 **	0.305 *	−0.311 *	−0.319 *
		Sig. (2-tailed)	0.000	0.101	0.389	0.905	0.949	0.984	0.000	0.035	0.031	0.027
	**Objective**	Pearson Correlation		−0.187	0.095	−0.015	0.011	0.047	0.612 **	0.292 *	−0.299 *	−0.300 *
		Sig. (2-tailed)		0.199	0.517	0.932	0.941	0.752	0.000	0.044	0.039	0.038
	**Difference**	Pearson Correlation			0.076	0.086	−0.048	−0.100	0.325 *	0.032	−0.030	−0.044
		Sig. (2-tailed)			0.603	0.625	0.745	0.497	0.023	0.828	0.841	0.765
**Refractive Error**		Pearson Correlation				−0.202	0.086	0.069	0.268	0.023	0.018	0.029
		Sig. (2-tailed)				0.244	0.555	0.643	0.063	0.879	0.904	0.843
**Tolerance to blur**		Pearson Correlation					0.008	−0.186	−0.136	0.203	−0.260	−0.265
		Sig. (2-tailed)					0.964	0.293	0.437	0.249	0.137	0.130
**Ocular Aberrations**	**Higher order**	Pearson Correlation						0.460 **	−0.042	0.117	−0.026	−0.045
		Sig. (2-tailed)						0.001	0.774	0.429	0.863	0.760
	**Average spherical**	Pearson Correlation							−0.053	−0.021	−0.015	0.001
		Sig. (2-tailed)							0.719	0.889	0.919	0.993
**Age**		Pearson Correlation								−0.042	0.000	0.002
		Sig. (2-tailed)								0.776	0.998	0.987
**Pupil size**		Pearson Correlation									−0.885 **	−0.898 **
		Sig. (2-tailed)									0.000	0.000
**DoF modelling**	**50% (max accom)**	Pearson Correlation										0.995 **
		Sig. (2-tailed)										0.000
